# Beneficial Effects of Standardized Extracts from Wastes of Red Oranges and Olive Leaves

**DOI:** 10.3390/antiox11081496

**Published:** 2022-07-30

**Authors:** Ilaria Burò, Valeria Consoli, Angela Castellano, Luca Vanella, Valeria Sorrenti

**Affiliations:** 1 Department of Drug and Health Science, University of Catania, 95125 Catania, Italy; ilariaburo95@gmail.com (I.B.); valeria_consoli@yahoo.it (V.C.); lvanella@unict.it (L.V.); 2 Mediterranean Nutraceutical Extracts (Medinutrex), Via Vincenzo Giuffrida 202, 95128 Catania, Italy; info@medinutrex.com; 3 CERNUT-Research Centre on Nutraceuticals and Health Products, University of Catania, 95125 Catania, Italy

**Keywords:** nutraceuticals, waste products, agri-food products, hepatic steatosis, heme oxygenase

## Abstract

The awareness of the large amount of waste produced along the food chain, starting in the agricultural sector and continuing across industrial transformation to the domestic context, has in recent years also aroused strong concern amongst the public, who are ing about the possible consequences that this could have on environmental sustainability, resource waste and human health. The aim of the present research is the recovery of substances with high added value from waste and by-products typical of the Mediterranean area, such as the residue from the industrial processing of red oranges, called *pastazzo* (peels, pulps and seeds), which is particularly rich in anthocyanins, flavanones and hydroxycinnamic acids, and has numerous nutraceutical properties, as well as the olive leaves coming from olive-tree pruning, which are rich in substances such as oleuropein, elenolic acid, hydroxytyrosol, tyrosol and rutin. The effect of Red Orange Extract (ROE) and Olive Leaf Extract (OLE) on HepG2 fatty storage capacity was assessed performing Oil Red O’ staining, and antioxidant properties of the extracts were evaluated following the steatosis model onset. Based on the results obtained, the preparation of natural extracts that are derived from these waste products can be useful for preventing, counteracting or delaying the onset of the complications of fatty liver disease, such as hepatic steatosis.

## 1. Introduction

Today, attention to lifestyle, including diet, leads to a high consumption of fruit and vegetables, as many people around the world follow a vegetarian diet. Consequently, this high consumption of fruit and vegetables leads to a substantial production of waste.

The awareness of the large amount of waste produced along the food chain, starting in the agricultural sector and continuing across industrial transformation to the domestic context, has in recent years also aroused strong concern amongst the public, who are worried about the possible consequences that this could have on environmental sustainability, resource waste and human health. The Waste Framework Directive (2008/98/EC) defines the criteria for managing food waste and establishes that the “by-product” can still be used in the industrial sector.

Researchers have recently paid a lot of attention to the development of innovative methods for recycling wastes. In waste management, it is essential to follow the circular economy approach, which, as a result of several factors [1], is becoming increasingly important and which represents a sustainable solution for using waste as valuable materials [2]. By-products generated by fruit- and vegetable-processing industries, represent a rich source of bioactive compounds. Recently, as an alternative method of using waste, many studies have extracted bioactive compounds from fruit and vegetable waste [1,2,3,4,5].

With a view to environmental sustainability and in the context of the circular economy, the recovery of nutrients and bioactive molecules obtained from the waste resulting from production chains is fundamental for the development of functional products with high added value that can be used in various production sectors, such as nutrition, nutraceuticals, and cosmetics. In Italy, the main agri-food production typical of the Mediterranean area, such as orange processing factories and the olive industry, generate large amounts of waste, which may be related to the polluting effects on soil and water and, if not recycled, represent a huge amount of raw material to be disposed of, resulting in high costs.

However, the by-products and the waste which comes from processing red oranges and olive leaves are particularly rich in bioactive compounds such as *a)* anthocyanins, flavanones and hydroxycinnamic acids, and *b)* oleuropein, elenolic acid, hydroxytyrosol, tyrosol and rutin, respectively, and have numerous nutraceutical properties such as the ability to control blood pressure, blood sugar content, triglyceride and cholesterol levels and so on [6,7,8,9,10,11]. So, the preparation of natural extracts may represent a way to use the waste and by-products of agricultural production by giving orange processing factories and the olive industry new possibilities that allow them to increase the added value of their production [12].

In particular, this research focuses its attention on the by-products and processing waste named *pastazzo* (peels, pulps and seeds) which comes from the processing of red oranges, and the olive leaves coming from olive-tree pruning.

The aim of the present research is the recovery of substances with high added value from waste, by-products and production surpluses for the preparation of natural extracts which, thanks to the effect of antioxidants naturally present, can be useful for preventing, counteracting or delaying the onset of hepatic steatosis complications. 

## 2. Materials and Methods

The powdered RED ORANGE EXTRACT (ROE) (batch no. 6/21) employed in this study was produced by Medinutrex (Catania, Italy) and was standardized to have ≥1.00% total anthocyanins, ≥1.00% total hydroxycinnamic acids and ≥10.00% total flavanones (determined by HPLC methods). It was used as ROE for the assays. The powdered OLIVE LEAF EXTRACT (OLE) (batch no. E21/2691) employed in this study was produced by Medinutrex (Catania, Italy) and was standardized to have ≥3.00% total polyphenols and ≥0.1% oleuropein (determined by the colorimetric method and HPLC method, respectively). It was used as OLE for the assays.

### 2.1. Industrial Extraction

Briefly, the industrial process for the preparation of both extracts (ROE and OLE) consists of an absorption phase of the aqueous filtered red orange and olive leaf solutions coming from the pressing of peels and pulps of red oranges and the solid/liquid extraction of olive leaves on adsorbent polymeric resins, followed by elution using hydro-alcoholic solutions. After the recovery of alcohol (ethanol), the concentrated aqueous extracts were spray-dried to obtain the powdered standardized extracts used in this study.

### 2.2. HPLC–PDA Analyses of Anthocyanins, Hydroxycinnamic Acids and Flavanones of ROE

Total anthocyanins were quantified as cyanidin 3-glucoside equivalents (g/100 g of ROE) and were analyzed by HPLC, according to Fabroni et al. [13]. Total hydroxycinnamic acids were quantified as ferulic acid equivalents (g/100 g of ROE) and were analyzed by HPLC, according to Rapisarda, et al. [14]. Total flavanones were quantified as hesperidin equivalents (g/100 g of ROE) and were analyzed by HPLC, according to Rouseff, et al. [15]. Briefly, for every HPLC analysis, 0.1 g of ROE was weighed and 10 mL of an acidic methanolic solution (0.1% HCl) was added. The solution was filtered through a 0.45 µM membrane filter and then injected directly into the HPLC system.

### 2.3. Determination of Total Polyphenols Content of OLE

The Folin–Ciocalteu colorimetric method [16] was applied for the determination of total polyphenols content of OLE. The total phenolic content was quantified as g of gallic acid equivalents (GAE)/100 g of OLE.

### 2.4. HPLC–PDA Analysis of Oleuropein of OLE

Oleuropein content (g/100 g of OLE) was analyzed by HPLC, according to D’Antuono, et al. [17] with slight modifications. Briefly. For the HPLC analysis of OLE, 0.1 g of extract was weighed, and 10 mL of methanol were added. The solution was filtered through a 0.45 µM membrane filter and then injected directly into the HPLC system.

### 2.5. Inhibition of DPPH

The free radical scavenging activity of ROE and OLE was evaluated using the DPPH (2,2-75 diphenyl-1-picrylhydrazyl) test. The reaction mixtures contained 86 μM DPPH, solubilized in ethanol, and different concentrations of ROE (0.01–0.05–0.1– 0.5–1–3– 6–9–11 mg/mL) and OLE (0.01–0.05–0.1–0.5–1–1.5–3–6 mg/mL), solubilized in a solution 1:5 ethanol/water. After 10 min at room temperature the absorbance at λ = 517 nm was recorded.

### 2.6. Cell Culture and Cell Viability Assay

Human liver cancer cell line (HepG2) was bought from American Type Culture Collection (ATCC, HB-8065, Rockville, MD, USA). Cells were cultured in Dulbecco’s modified Eagle’s medium (DMEM) 1 g/L glucose, supplemented with 10% FBS, 1% penicillin and streptomycin solution and maintained at 37 °C and 5% CO_2_. HepG2 cells were seeded at a concentration of 2 × 10^5^ cells per well of a 96-well, flat-bottomed microplate. The cultures were maintained in the absence or presence of the different concentrations of ROE and OLE reported above for 72 h. Cell viability was determined by MTT assay, measuring the activity of cellular enzymes that reduce the tetrazolium dye, 3-(4,5-dimethylthiazol-2-yl)-2,5-diphenyltetrazolium bromide, a yellow tetrazole (MTT), to its insoluble formazan, giving a purple colour in living cells. MTT solution was added to cells and maintained for 2 h, then formazan produced by viable cells was dissolved in DMSO and absorbance was detected at λ = 570 nm in a microplate reader (Biotek Synergy-HT, Winooski, VT, USA). Eight replicate wells were used for each group, and at least two separate experiments were performed.

### 2.7. Cellular Model of Hepatic Steatosis

For the purpose of creating an *in vitro* model of hepatic steatosis, HepG2 cells were seeded in a 96-well cell culture plate and maintained for 24 h with Dulbecco’s modified Eagle’s medium (DMEM) 1 g/L glucose, supplemented with 10% FBS, 1% penicillin and streptomycin solution. Then, the medium was changed to DMEM integrated with Free Fatty Acids (FFA) (oleic acid and palmitic acid 2:1) 0.75–1 mM and incubated for 72 h to induce lipid droplets accumulation.

### 2.8. Determination of Heme Oxygenase-1 (HO-1) Levels (ELISA)

In order to evaluate the effect of ROE and OLE on HO-1 levels, HepG2 cells were maintained with different concentration of each extract for 24 h (0.01–0.05–0.1–0.5–1–1.5– 3–6 mg/mL). Moreover, HO-1 levels were measured after treatment with 0.75 mM of FFA in the presence or absence of 1:1 combination of different concentrations of OLE and ROE (0.1:0.1 mg/mL; 0.25:0.25 mg/mL; 0.5:0.5 mg/mL) for 72 h.

HO-1 levels were assessed on cell lysates using Simple Step ELISA (ab207621, Abcam, Cambridge, UK), according to the manufacturer’s instructions. The absorbance was measured at λ = 450 nm using a microplate reader. The experiment was performed at least three times and the results are expressed as pg/mL.

### 2.9. Oil Red O’Staining

Staining was performed using 0.21% Oil Red O in 100% isopropanol (Sigma-Aldrich, St. Louis, MO, USA). After treatment, HepG2 cells were fixed in 10% formaldehyde and stained with Oil Red O for 10 min, rinsed with 60% isopropanol (Sigma-Aldrich, St. Louis, MO, USA), and the Oil Red O eluted by adding 100% isopropanol for 10 min, then optical density (OD) was measured at λ = 490 nm. Lipid droplets accumulation was examined and figures were taken using an inverted multichannel LED fluorescence microscope (Evos, Life Technologies, Grand Island, NY, USA).

### 2.10. Measurement of Mitochondrial Membrane Potential

Following a 6h co-treatment of FFA 0.75 mM and combinations of OLE and ROE, cells were incubated with a 3 μM JC-1 staining solution (T3168, Invitrogen, Waltham, MA, USA) at 37 °C for 20 min and then washed with PBS in order to visualize the fluorescence with a fluorescence microscope (EVOS Fl AMG). The JC-1 probe aggregates to form a polymer in the mitochondrial matrix of healthy cells, producing a strong red fluorescence (Ex = 585 nm, Em = 590 nm). Otherwise, dysfunctional mitochondria present JC-1 monomers, resulting in the emission of a green fluorescent signal (Ex = 514 nm, Em = 529 nm). The results are expressed as the ratio of red/green fluorescence.

### 2.11. Thiol (RSH) Group Determination

The measurement of thiol groups (RSH) concentration was performed as it reflects almost 90% of GSH cellular content. The assay is based on a reaction of thiol groups with 2,2-dithio-bis-nitrobenzoic acid (DTNB). The assay mixture, containing samples and DTNB, was incubated in the dark at room temperature for 20 min and finally centrifuged at 3000 rpm for 10 min. The supernatant was collected and set in a black 96-well plate to measure the absorbance in a microplate reader at λ = 412 nm. The experiment was performed at least three times and the results are expressed as pmoles/µL.

### 2.12. Measurement of Lipid Peroxidation

HepG2 cells were treated with FFA in the presence or absence of a combination of different concentrations of OLE and ROE (0.1:0.1 mg/mL; 0.25:0.25 mg/mL; 0.5:0.5 mg/mL). The amount of Fe^3+^, derived from the oxidation of Fe^2+^ in the presence of xylenol orange, was correlated with LOOH levels.

The assay was conducted by adding to 200 μg of total cell lysate, 100 μM xylenol orange, 250 μM ammonium ferrous sulphate, 90% ethanol, 4 mM butylated hydroxytoluene and 25 mM H_2_SO_4_. Samples were incubated at room temperature for 30 min, and the absorbance was measured at λ = 560 nm using a microplate reader (Biotek Synergy-HT, Winooski, VT, USA). Calibration was performed using hydrogen peroxide (0.2–20 μM). The experiments were conducted at least three times and the results were expressed as a percentage of control.

### 2.13. Measurement of HMG-CoA Reductase Activity

The capacity of ROE and OLE to modulate the activity of purified HMG-CoA reductase was measured by HMG-CoA Reductase Activity Assay Kit from Abcam (Cambridge, UK). It is a colorimetric method based on the consumption of NADPH by the enzyme, which can be measured by the decrease in absorbance at λ = 340 nm. It was tested using 0.5 mg/mL concentration for each extract and a 1:1 combination of them. The results are expressed as a percentage of inhibition of HMG-CoA reductase activity and represent data obtained from four experimental determinations.

### 2.14. Statistical Analysis

At least three independent experiments were performed for each analysis. The statistical significance (*p* < 0.05) of the differences between the experimental groups was determined using Fisher’s method for analyses of multiple comparisons. For comparison between treatment groups, the null hypothesis was tested by either a single-factor analysis of variance (ANOVA) for multiple groups or an unpaired t-test for two groups, and the data are presented as means ± SEM.

## 3. Results

### 3.1. Chemical Comosition of ROE and OLE

The extracts (ROE and OLE), obtained from the initial vegetable matrices consisting of peels and pulps coming from the industrial processing of red oranges and olive leaves coming from the pruning of olive trees, were analytically characterized with respect to the total anthocyanins, hydroxycinnamic acids and flavanones contents for ROE and to total polyphenols and oleuropein content for OLE, respectively. ROE and OLE were characterized through HPLC–PDA for their individual and total content of bioactive compounds. The results of individual quantification of anthocyanins in ROE showed that cyanidin 3-glucoside is the predominant anthocyanin, while the quantification of hydroxycinnamic acids showed that the most abundant hydroxycinnamic acid in ROE is ferulic acid. Moreover, the flavanones’ pattern of ROE showed that the major flavanone compound is hesperidin. Regarding OLE, it was shown to be rich in total polyphenols, with oleuropein being the main phenolic compound in the extract (Table 1).

### 3.2. Free Radical Scavenging Activity of ROE and OLE

**Figure 1:**
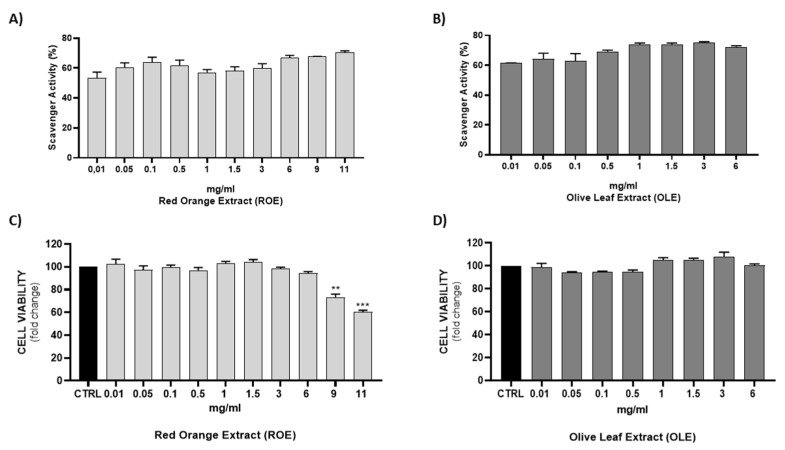
DPPH radical scavenging activity of (**A**) ROE and (**B**) OLE at different concentrations. Results are expressed as a percentage of inhibition. Evaluation of HepG2 cell viability in the absence or presence of (**C**) ROE and (**D**) OLE at different concentrations for 72 h. Results are expressed as mean ± SEM. Significant vs CTRL: ** *p* < 0.005; *** *p* < 0.0005.

The anti-radical activity of this extract was evaluated with the DPPH assay. The results of DPPH assay are shown in Figure 1A,B ROE and OLE were tested at different concentrations: 0.01–0.05–0.1–0.5–1–3–6–9–11 mg/mL and 0.01–0.05–0.1–0.5–1–1.5–3–6 mg/mL, respectively. ROE and OLE displayed a strong dose-dependent radical scavenging effect, which reached about 75% inhibition at the highest concentrations.

### 3.3. Effect of ROE and OLE on Cell Viability

MTT cell-proliferation assay measures the reduction of a tetrazolium component (MTT) into an insoluble formazan product by the mitochondria of viable cells. The MTT assay is a quantitative and sensitive detection of cell viability as it measures the growth rate of cells by virtue of a linear relationship between cell activity and absorbance. Figure 1C,D shows that the highest tested concentrations (9–11 mg/mL) of ROE exhibited a strong reduction of viable HepG2 cells. Contrarily, no significant differences were observed with lower concentrations of ROE compared to the control group and with all tested concentrations of OLE, indicating a slight in vitro cytotoxic activity.

### 3.4. Antioxidant Activity of ROE and OLE Mediated by HO-1 Induction

**Figure 2:**
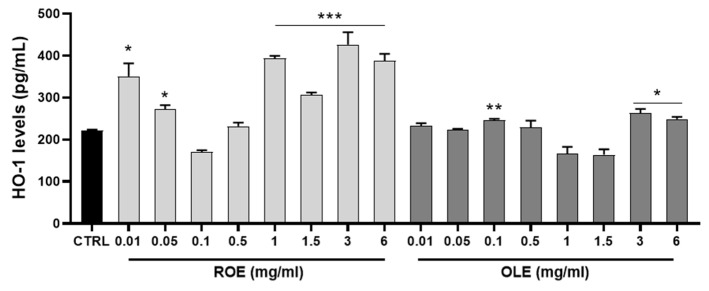
Measurement of HO-1 levels after 24 h of treatment with ROE and OLE at different concentrations. Results are expressed as mean ± SEM. Significant vs CTRL: * *p* < 0.05; ** *p* < 0.005; *** *p* < 0.0005.

HO-1 levels were examined using ELISA assay to quantify total endogenous cellular protein after the treatment for 24 h of HepG2 cells with different concentrations of ROE and OLE (0.01–0.05–0.1–0.5–1–1.5–3–6 mg/mL). This cell-based method served as rapid screening to select extract concentrations with the better HO-1 inducer profile. The results obtained in our experimental conditions showed that ROE, in a dose-dependent manner, and OLE, only at higher concentrations, were able to induce HO-1 protein (Figure 2).

### 3.5. Effect of ROE and OLE on HepG2 Fatty Storage

**Figure 3:**
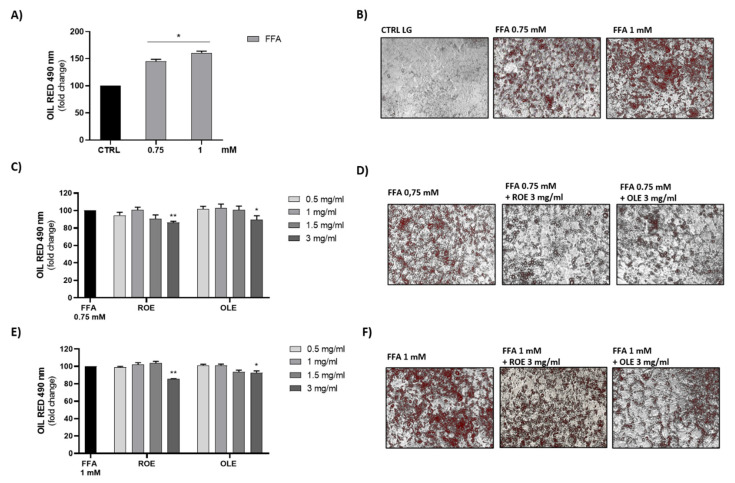
(**A**) Evaluation of lipid droplets formation after 72 h of treatment with FFA (0.75–1 mM) and (**B**) representative images of Oil Red O’ staining on steatosis model. Results are expressed as mean ± SEM. Significant vs CTRL: * *p* < 0.05. (**C**,**E**) Effect of ROE and OLE treatments on lipid droplets accumulation in presence of FFA 0.75 mM and 1 mM. Results are expressed as mean ± SEM. Significant vs FFA (0.75 mM): * *p* < 0.05; ** *p* < 0.005. Significant vs FFA (1 mM): * *p* < 0.05; ** *p* < 0.005. (**D**,**F**) Representative images of Oil Red O’ staining on HepG2 treated for 72 h with FFA 0.75 Mm and 1 mM in the absence or presence of a higher concentration (3 mg/mL) of each extract.

Human liver cells (HepG2) treated with FFA were used as a model of hepatic steatosis. As shown in Figure 3A,B, increasing concentrations of FFA (0.75–1 mM) caused an increasing accumulation of lipid droplets. Only at a concentration of 3 mg/mL were both OLE and ROE able to reduce the lipid drops accumulation caused by the treatment of HepG2 cells with 0.75 and 1 mM FFA, while at the lowest concentrations this effect was not evident (Figure 3C–F).

**Figure 4:**
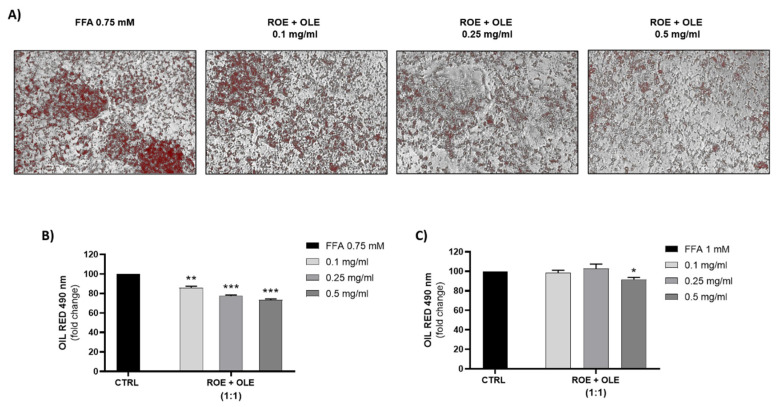
(**A**) Representative images of Oil Red O’ staining on HepG2 treated for 72 h with FFA 0.75 mM and different 1:1 combination of ROE and OLE (**B**,**C**). Effect of 1:1 combinations of ROE and OLE on lipid droplets accumulation in the presence of FFA 0.75 mM and 1 mM. Significant vs CTRL: * *p* < 0.05; ** *p* < 0.005; *** *p* < 0.0005.

Since only high extract concentrations (3mg/mL) were able to reduce lipid accumulation, we decided to investigate if the co-treatment of HepG2 cells with the lower concentrations of the two extracts could have a synergistic effect. We measured the lipid accumulation obtained after treatment with a combination of different concentrations of OLE and ROE (0.1:0.1 mg/mL; 0.25:0.25 mg/mL; 0.5:0.5 mg/mL) (Figure 4). All concentrations tested significantly reduced lipid droplet accumulation obtained with 0.75 mM FFA but not those obtained with 1 mM FFA. Therefore 0.75 mM FFA concentration compared to 1 mM FFA could represent the best condition to further investigate molecular mechanisms involved in the beneficial effect of treatment with a combination of different concentrations of OLE and ROE (0.1:0.1 mg/mL; 0.25:0.25 mg/mL; 0.5:0.5 mg/mL) in preventing lipid accumulation.

### 3.6. Effect of ROE and OLE on Oxidative Damage and Mitochondrial Dysfunction

**Figure 5:**
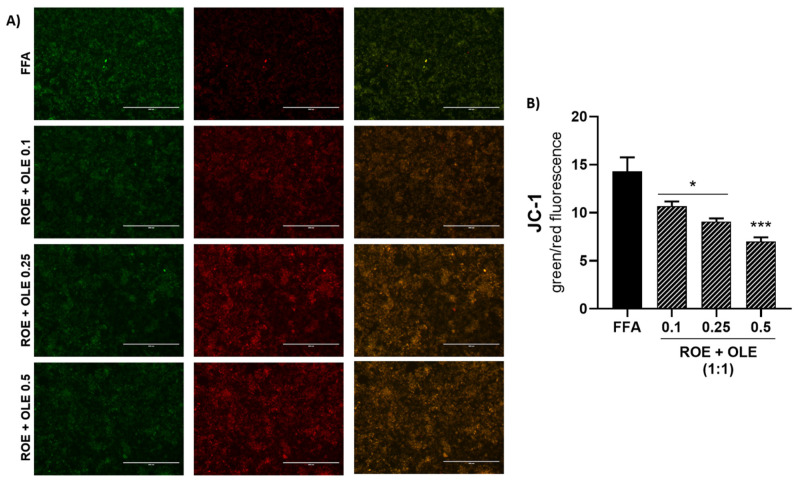
(**A**) Fluorescence images of JC-1 staining after 6 h of treatment. (**B**) Effect of ROE and OLE combinations on mitochondrial membrane potential. Significant vs FFA: * *p* < 0.05; *** *p* < 0.0005.

The low signal of JC-1 dimer (red fluorescence) and a higher presence of JC-1 monomer (green fluorescence) in FFA treated cells indicated oxidative damage and mitochondrial dysfunction, as already reported [18]. As shown in Figure 5, the co-treatment of FFA 0.75 mM and combinations of OLE and ROE (0.1:0.1 mg/mL; 0.25:0.25 mg/mL; 0.5:0.5 mg/mL) were able to reduce the ratio of red/green fluorescence signal in a dose-dependent manner, highlighting a protective effect of the extracts against FFA-induced oxidative stress. 

### 3.7. Effect of ROE and OLE on Thiol (RSH) Group Levels

**Figure 6:**
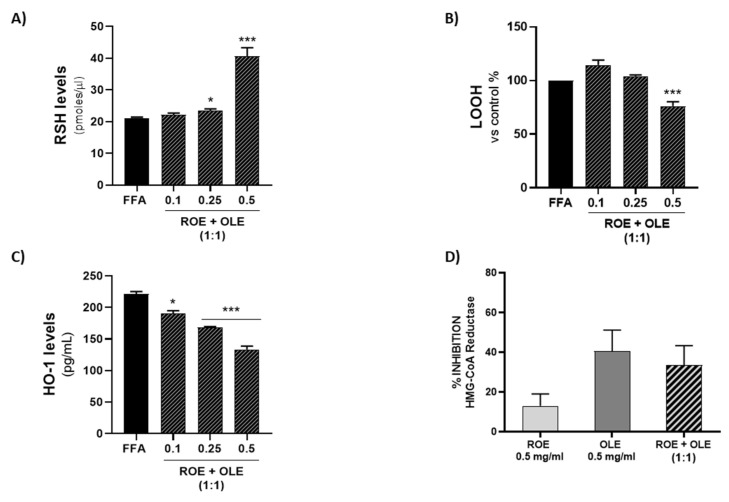
Evaluation after 72 h of different 1:1 combination of ROE and OLE effect on (**A**) RSH levels; (**B**) LOOH; (**C**) HO-1 levels. Significant vs FFA: * *p* < 0.05; *** *p* < 0.0005. (**D**) Measurement of HMG-CoA reductase inhibiting activity of ROE and OLE (0.5 mg/mL) and 1:1 combination of them. Results are expressed as mean ± SEM.

In order to evaluate RSH cellular content, thiol group determination via spectrophotometric assay was performed. Our results showed a dose-dependent increase in reduced RSH after 72 h of treatment with 0.75 mM FFA (Figure 6A) in the presence of a combination of different concentrations of OLE and ROE (0.1:0.1 mg/mL; 0.25:0.25 mg/mL; 0.5:0.5 mg/mL). At 0.5:0.5 mg/mL concentration, a significant synergistic effect was produced.

### 3.8. Effect of ROE and OLE on LOOH Levels

In order to evaluate lipid peroxidation levels, LOOH determination via spectrophotometric assay was performed. Our results showed a decrease in LOOH levels after 72 h of treatment with 0.75 mM FFA (Figure 6B) in the presence of a combination of different concentrations of OLE and ROE (0.1:0.1 mg/mL; 0.25:0.25 mg/mL; 0.5:0.5 mg/mL). At 0.5:0.5 mg/mL concentration, a significant synergistic effect was produced.

### 3.9. Effect of ROE and OLE on HO-1 Expression in Steatosis Model

In order to evaluate HO-1 protein expression, HO-1 determination by ELISA assay was performed. Our results showed a decrease in HO-1 levels after 72 h of treatment with 0.75 mM FFA (Figure 6C) in the presence of a combination of different concentrations of OLE and ROE (0.1:0.1 mg/mL; 0.25:0.25 mg/mL; 0.5:0.5 mg/mL). At 0.5:0.5 mg/mL concentration, a significant synergistic effect was produced.

### 3.10. Effect of ROE and OLE on HMG-CoA Reductase Activity

The results obtained in our experimental conditions showed that OLE displayed a strong inhibitory effect on HMG-CoA reductase activity, which reached about 40% inhibition at the concentration of 0.5 mg/mL, while the effect of ROE at the same concentration was lower (about 15%). The combination of OLE and ROE showed that the inhibitory effect of OLE prevailed over the moderate inhibitory effect of ROE (Figure 6D). Therefore, the ability of ROE, but especially of OLE, to inhibit the activity of HMG CoA reductase supports their use as an alternative cholesterol-lowering agents to statins.

## 4. Discussion

The extraction of bioactive compounds from food industry by-products can be helpful in the prevention of various diseases, thanks to the formulation of nutraceuticals or to the development of functional foods.

Antioxidant activity and other health-beneficial effects of bioactive compounds present in most food and medicinal herbs have been reported [19]. Phenolic compounds present in edible sources retard the oxidative degradation of lipids by direct quenching of ROS [20]. Molecular mechanisms of phenolic bioactive compounds of edible plants that have healthful effects are also associated with antioxidant and anti-inflammatory effects involving HO-1 induction [21]. HO-1 has been reported to play an important role in antioxidative stress and cytoprotective systems in the liver. Once expressed, HO-1 catalyzes the degradation of heme to yield equimolar amounts of three end-products: biliverdin, CO and ferrous iron [22], which perform anti-inflammatory and antioxidant activities. Particularly, biliverdin is transformed into bilirubin, which functions as an antioxidant and vasoactive molecule [23].

Many researchers have reported that natural extracts obtained from the waste products of the processing factories have a free radical scavenging and potent antioxidant activity [24,25,26]. However, although some authors have obtained extracts from wastes of oranges or olive leaves, the chemical characterization of the bioactive compounds was obtained only after a simple lab-scale extraction [8,9].

In the present study, red oranges and olive leaf waste was used as starting material for the production of standardized extracts that were obtained with an industrial process of the extraction and recovery of bioactive compounds on adsorbent resins from by-products, following by a spray-drying process that does not require the use of organic solvents.

ROE and OLE showed antioxidant activities in a concentration-dependent manner as shown for both the DPPH test and HO-1 ELISA assay. Moreover, data obtained with MTT cell viability assay, indicating a slight in vitro cytotoxic activity, support studies reporting that many of the natural compounds show the ability to induce Nrf2/HO-1 axis without cytotoxic effects. Non-alcoholic fatty liver disease (NAFLD) is a common condition caused by the storage of extra fat in the liver, resulting in organ damage. NAFLD is increasingly common around the world, especially in Western nations. NAFLD can progress to non-alcoholic steatohepatitis (NASH) or steatosis, both characterized by increased oxidative stress and inflammation. NASH, in turn, can lead to cirrhosis or to related complications, such as hepatocellular carcinoma. However, it is possible to prevent or even reverse fatty liver disease with lifestyle changes [7].

HepG2 cells have been widely used in models of hepatic steatosis in vitro [27,28,29]. In our experimental conditions, FFA-exposed HepG2 cells showed a dose-dependent increase in accumulation of lipid droplets.

However, since only high extract concentrations (3 mg/mL) were able to reduce lipid accumulation, we decided to investigate if the co-treatment of HepG2 cells with lower concentrations of the two extracts could have a synergistic effect. All lower concentrations tested significantly reduced lipid droplet accumulation obtained with 0.75 mM FFA but not those obtained with 1 mM FFA. Therefore, 0.75 mM FFA concentration could represent the best condition to further investigate molecular mechanisms involved in the beneficial effect of OLE and ROE in preventing lipid accumulation.

To further identify the possible biochemical mechanisms underlying ROE and OLE antioxidant effects, we also investigated the ability of bioactive compounds contained in ROE and OLE to enhance endogenous antioxidant defences in HepG2 cells in the presence of FFA. It has been reported that glutathione depletion is considered a potential biomarker of FFA-induced hepatotoxicity [28]. The ability of ROE and OLE, in HepG2 cells, to increase RSH levels is related to a decrease in oxidative stress, reflected by lowering JC-1 ratio of red/green fluorescence signal, by LOOH reduction and to significantly reduced lipid droplet accumulation. Moreover, the decrease in oxidative stress is related to a significant reduction in the HO-1 expression in HepG2 cells treated with FFA in the presence of the combination of ROE and OLE. It is known that HO-1 is strongly induced under cellular stressful conditions and its expression is transcriptionally regulated by the Nrf2/HO-1 axis [30,31]. Therefore, since the co-treatment of HepG2 cells with FFA and ROE plus OLE is able to reduce the formation of LOOH and increase the endogenous antioxidant defences, the Nrf2/OH-1 axis activated by FFA treatment is not activated in the presence of the two extracts.

Hepatic lipotoxicity implies that accumulation, not only of lipotoxic molecules such as free fatty acids (FFAs) and their derivatives, but also of cholesterol [32,33,34] within hepatic cells, may directly cause cellular toxicity or act in a proinflammatory or profibrotic manner [35]. It has been reported that, in humans, increased intake of saturated fat may influence LDL cholesterol levels [36]. Dongiovanni et al. reported that use of cholesterol-lowering drugs, HMG-CoA reductase inhibitors known as statins, was associated with significant protection from steatosis, inflammation, and fibrosis [37]. However, studies by Mancini et al. 2011 support the use of HMG-CoA reductase inhibitors from natural sources due to the sides effects of statins [38]. Therefore, we examined whether OLE and ROE at a concentration of 0.5 mg/mL were able not only to reduce the accumulation of lipids in HepG2 cells but also to reduce the activity of HMG-CoA reductase.

In our experimental conditions the ability of ROE, but especially of OLE, to inhibit the activity of HMG-CoA reductase supports their use as an alternative cholesterol-lowering agents to statins.

## 5. Conclusions

The extracts obtained in the present research (ROE and OLE), coming from the residue of the processing of red oranges (*pastazzo*) and olive leaves, are rich in bioactive compounds such as polyphenols, have a good antioxidant capacity, are able to reduce the accumulation of free fatty acids and could act as cholesterol-lowering agents. The synergistic effect highlighted by the co-treatment of HepG2 cells with the two extracts (ROE and OLE) allows us to hypothesize that the preparation of a new nutraceutical formulation deriving from the combination of both extracts shall enhance their antioxidant effect and can be useful in preventing, counteracting or delaying the onset of the complications of hepatic steatosis. Furthermore, ROE and OLE obtained from the waste and by-products of agricultural production could represent a solution not only to emphasize these sources but also to give red orange processing factories and the olive industry new possibilities that allow them to increase the added value of their production.

## Figures and Tables

**Table 1:** Chemical composition of ROE and OLE used in this study. Results are expressed as: ^a^ g of gallic acid equivalents (GAE)/100 g of OLE; ^b^ g of oleuropein equivalents/100 g of OLE; ^c^ g of cyanidin 3-glucoside equivalents/100g of ROE; ^d^ g of ferulic acid equivalents/100 g of ROE; ^e^ g of hesperidin equivalents/100 g of ROE.

Compounds	ROE	OLE
*Total Polyphenols*	*not determined*	*3.82 g/100 g ^a^*
Hydroxytyrosol	absent	0.10 g/100 g
Tyrosol	absent	0.05 g/100 g
Oleuropein	absent	0.40 g/100 g
*Total Phenols*	*absent*	*0.55 g/100 g ^b^*
Cyanidin 3,5-diglucoside	0.06 g/100 g	absent
Delphinidin 3-glucoside	0.02 g/100 g	absent
Cyanidin 3-glucoside	0.31 g/100 g	absent
Cyanidin 3-rutinoside	0.02 g/100 g	absent
Petunidin 3-glucoside	0.02 g/100 g	absent
Delphinidin 3-(6″-malonyl)glucoside	0.02 g/100 g	absent
Peonidin 3-glucoside	0.02 g/100 g	absent
Petunidin 3-(6″-malonyl)glucoside	0.03 g/100 g	absent
Cyanidin derivative	0.03 g/100 g	absent
Cyanidin 3-(6″-malonyl)glucoside	0.27 g/100 g	absent
Cyanidin 3-(6″-dioxalyl)glucoside	0.06 g/100 g	absent
Pelargonidin derivative	0.02 g/100 g	absent
Peonidin 3-(6″-malonyl)glucoside	0.02 g/100 g	absent
Cyanidin derivative	0.03 g/100 g	absent
Peonidin derivative	0.01 g/100 g	absent
Cyanidin derivative	0.03 g/100 g	absent
Cyanidin derivative	0.02 g/100 g	absent
Cyanidin derivative	0.03 g/100 g	absent
*Total Anthocyanins*	1.02 g/100 g ^c^	*absent*
*p*-Coumaric acid	0.17 g/100 g	absent
Ferulic acid	0.62 g/100 g	absent
Sinapic acid	0.24 g/100 g	absent
*Total Hydroxycinnamic Acids*	1.03 g/100 g ^d^	*absent*
Narirutin	3.17 g/100 g	absent
Hesperidin	10.1 g/100 g	absent
Didymin	1.13 g/100 g	absent
*Total Flavanones*	14.4 g/100 g ^e^	absent

## Data Availability

Not applicable.
